# Validation and usability of SeDAR e-health video for enhancing cervical cancer screening

**DOI:** 10.1371/journal.pone.0310555

**Published:** 2024-09-17

**Authors:** Rodziah Romli, Emma Mirza Wati Mohamad, Rahana Abd Rahman, Kah Teik Chew, Syahnaz Mohd Hashim, Azmawati Mohammed Nawi

**Affiliations:** 1 Faculty of Medicine, Department of Public Health Medicine, Universiti Kebangsaan Malaysia, Cheras, Kuala Lumpur, Malaysia; 2 Institut Latihan Kementerian Kesihatan Malaysia Alor Setar, Ministry of Health, Alor Setar, Malaysia; 3 Faculty of Social Sciences and Humanities, Centre for Research in Media and Communication (MENTION), Universiti Kebangsaan Malaysia, Bangi, Selangor, Malaysia; 4 Faculty of Medicine, Department of Obstetrics and Gynaecology, Universiti Kebangsaan Malaysia, Cheras, Kuala Lumpur, Malaysia; 5 Faculty of Medicine, Department of Family Medicine, Universiti Kebangsaan Malaysia, Cheras, Kuala Lumpur, Malaysia; Hamadan University of Medical Sciences School of Dentistry, ISLAMIC REPUBLIC OF IRAN

## Abstract

**Background:**

The cervical cancer (CC) incidence rate is increasing among young women aged <50 years despite early screening is proven effective. Electronic health (e-health) has great potential for disseminating health education.

**Methods:**

This study validated a newly developed e-health tool “SeDAR^®”^ and assessed its usability via evaluations by health experts (HE), media experts (ME), and women. The SeDAR^®^ content was developed based on protection motivation theory (PMT) using the nominal group technique and in-depth interviews that involved HE and women, respectively. Content validation was performed among the HE (n = 12) and ME (n = 5) using the content validation index (CVI) to identify their agreement. Subsequently, the Video Engagement Scale (VES^®^) was used to validate SeDAR^®^ among women of different ethnicities (n = 11) to achieve ecological validity. The experts and women also commented on the presentation of the video.

**Results:**

The validation yielded a good CVI among the HE (scale-level CVI-average [SCVI/Ave] = 0.986; scale-level CVI-universal agreement [SCVI/UA] = 0.900) and ME (SCVI/Ave = 0.979, SCVI/UA = 0.897). The highest VES^®^ score [mean (±SD) = 92.90(±3.46)] proved the ecological validity of SeDAR^®^. The experts’ feedback established that SeDAR^®^ conveyed a clear message about awareness of performing CC screening and was suitable for public viewing. The women considered SeDAR^®^ easy to understand, and it advised early exposure for early CC screening.

**Conclusions:**

SeDAR^®^ was valid and could constitute an important e-health tool to improve motivation and uptake of CC screening.

## Introduction

The cervical cancer (CC) incidence rate is increasing among young women aged <50 years, and numbers 14 cases per 100,000 women per year [1]. CC is associated with persistent human papillomavirus (HPV) infection via frequent sexual activity. Approximately 80% of women will be infected by HPV at some point in their lifetime, with >50% being infected when they are 20–24 years old [1,2]. The CC development process is slow, with abnormal changes of precancerous cells beginning with cervical intraepithelial neoplasia (CIN)1 to CIN3 and progressing to carcinoma in situ (Stage 0) before being diagnosed as Stage I cervical cancer. This development process spans 10–20 years starting from CIN1 [1–4]. Early transformation into CIN is typically asymptomatic; thus, women are unaware that their cervical cells are undergoing abnormal transformation.

The World Health Organization (WHO) formulated a global strategy to eliminate CC as a public health problem by the 21st century. The strategy guideline vision includes a threshold rate of an estimated age-standardised incidence rate (ASR) of 4.0 per 100,000 women, with 70% of women screened before the age of 35 years and re-screened before the age of 45 years [5,6]. However, the availability of subsidised free screening programmes at health clinics throughout Malaysia cannot guarantee the presence of women at CC screenings. The coverage of women undergoing CC screening in Malaysia within a 5-year period (2014–2018) remained at <40% (range: 23–26%), and only 36.6% of women underwent screening in 2019 [7]. This screening rate remains far from reaching the WHO recommendation of 70%. The CC screening is free subsidized to eligible Malaysian women does not guarantee attendance for screening if women do not have the knowledge and self-efficacy motivation to attend screening [8–11].

Past studies in Malaysia demonstrated that the barriers to CC screening were lack of awareness related to CC and CC screening [9] and the apathetic attitude of young women who considered themselves not at risk of CC [12]. Additionally, social stigma was a barrier to accessing health facilities among single unmarried women [8] and people without obstetrics and gynaecology needs [10]. These findings indicated that conservative and opportunistic approaches, which require women to attend health centres to obtain information from health professionals, are less accessible to marginalised women who lack access to health facilities.

Electronic health (e-health) education is an alternative means of disseminating health information [13]. E-health is an innovative means of promoting health education through prevention and can be achieved via entertainment-education strategies using multimedia and communication interventions [14]. E-health education is also a modern alternative for disseminating health information and educating women using multimedia. It is a more effective method to reach women and saves on the costs of printing and human resources as information intermediaries [13,15]. Currently, the developed countries use e-health interventions and media campaigns as an education strategy to reach more women and promote CC screening [16–19]. Investing in e-health tool development would enable the cultivation of knowledge on the benefits of CC screening and reach marginalised women. Health behaviour change interventions are presented in myriad forms and include videos [20,21], fotonovelas [14,17], and short films [22,23].

Motivational emphasis on lifelong regular screening is important to reduce the CC incidence rate. The e-health approach would motivate women with high knowledge and awareness levels regarding the importance of early CC screening to attend screening voluntarily [13,23,24]. Accordingly, the increased knowledge and awareness amongst women is expected to lead to increased motivation to attend CC screening programmes. The protection motivation theory (PMT) framework that includes seven constructs [perceived vulnerability, perceived severity, fear (threat appraisal), response cost (coping appraisal), response efficacy, self-efficacy, protection motivation (intention)] is used as a guideline to impart motivational aspects to newly designed e-health tools.

PMT is a social cognitive model that was described by Roger in 1975 to explain how an individual is motivated towards self-protection against health threats. PMT explains that a person’s intention to perform behaviour, such as CC screening, is based on two appraisal cognitive processes: threat and coping. When faced with a threat (CC), the person will first appraise it based on how likely it can affect them (perceived vulnerability), how bad it is (perceived severity), and its potential consequences (fear appraisal). Subsequently, the person decides how the threat can be reduced by determining how likely they could perform the required behaviour, i.e., CC screening (self-efficacy), evaluate the potential cost (response cost), and the resources for performing such behaviour (response efficacy) [25–28]. This theoretical framework is the most motivationally tailored intervention guidance [29], and previous studies proved the effectiveness of motivational focus in increasing CC screening uptake [28,30–33].

Hence, to motivate women from the youngest age group to improve their CC knowledge and undergo screening, this study developed a motivational e-health video titled “SeDAR^®^”. Guided by PMT [22], this study focused on the validation and usability of SeDAR^®^ among healthcare experts (HE) and media experts (ME) as providers, and women as users, respectively.

## Materials and methods

### Video content and appearance

This study was part of a larger study which funded and approved by the Universiti Kebangsaan Malaysia Medical Research Ethics Committee (grant number: FF-2021-499; date of approval: 28 October 2021). All participants give informed consent to participate in the entire SeDAR^®^ content development process starting from January 1, 2022 to September 1, 2022. We created the SeDAR^®^ content based on five combined input sources. The first input from systematic literature review (SLR) findings on e-health videos in previous studies [13]. The input regarding SLR was derived based on meta-analysis finding that demonstrated e-health tools was effective in increasing the CC screening uptake regardless geographical area or minority communities [13]. Therefore, health education through e-health tools has great potential to promote CC screening in the community at large regardless of geographic barriers. The finding highlighted the important of using appropriate frameworks, user engagement and culturally tailored to be prioritized in developing an effective e-health tool [13].

The second input was regarding the cross-sectional study among Malaysian women’s (n = 526) which demonstrated the motivational aspect towards CC screening based on PMT [11]. The finding highlighted the sexually active women, have heard and have undergone CC screening feel less threatened with low coping appraisals [11]. Undergoing CC screening made women perceived more response efficacy (p = 0.011), more self-efficacy (p<0.001) and higher protection motivation (p<0.001) towards CC screening [11]. We used these inputs as guideline to conduct focus group discussion (FGD) among multidisciplinary health practitioners and in-depth interview (IDI) among various ethnicities’ women in developing the content of SeDAR^®^.

The third input is from FGD among multidisciplinary health practitioners (n = 12) who involved in CC management, promotion, and screening [34]. We included the scene involving a doctor’s consultation during the CC screening counselling session to highlight the HE’s concern regarding the woman’s lack of knowledge. The script included simple, informative messages that focused on early diagnosis, early-stage CC being asymptomatic, and promoting regular screening without using medical jargon [34].

The forth input based on IDI with women (n = 7) of various ethnicities to understand their perspectives regarding screening. The finding highlight women’s misperception regarding CC screening [34]. From the finding, we highlighted the need for screening regardless of symptoms and marriage status and regular periodic screening. The IDI finding showed that women placed great emphasis on fulfilling their role in the family, ensuring their reproductive health, and were concerned about the CC screening procedure [34]. To address these aspects, the SeDAR^®^ storyline involves a woman with a caring husband, cheerful children, and supportive mother-in-law and friends who provide encouragement on the importance of CC screening.

The fifth input derived from group discussion sessions among ME with selected diverse eligible women (n = 10) to determine the e-health video material and appearance. The finding showed that women preferred the e-health tools in form of entertainment film video based on the real story of CC survivor.

Based on the provided five inputs, a local video production company ME with extensive experience produced the video storylines and script. The storyline and script were developed and the video was produced using an entertainment-education (narrative-based) approach [13,25,35]. Entertainment-education is an innovative approach that encourages the audience to appreciate the storyline and understand the motivation regarding the importance of the CC screening. The short film in the SeDAR^®^ video revolved around ordinary people to elicit changes in knowledge, attitude, and social norms among Malaysian women. All the names and characters played in this video are purely acting based on research findings. Actors /or their legal guardian(s) have granted the informed consent on the right to post, display, market and distribute the product, in whole or in part, alone or together with other products, for any purpose determined by the researcher. This grant includes the right to use their images in an online open-access publication, advertising or publicity purposes. The individual in this manuscript has given written informed consent (as outlined in PLOS consent form) to publish these case details.

“Sedar” is a Malay word that means “to realise”, and SeDAR^®^ refers to “**Se**rviks **D**isaring, **A**malan **R**utin” (“Screened Cervix, Routine Practice”). The SeDAR^®^ video is in Malay with a few simple English words commonly used by Malaysians. The subtitles are in both Malay and English. The SeDAR^®^ video seeks to address motivation-focused messages such as knowledge deficit (perceived vulnerability), early detection (perceived severity), and fear of being diagnosed (threat appraisal). The video focused on social support to encourage perceived self-efficacy, better quality of life as response efficacy, and undergoing CC screening either using the Pap smear or HPV test as coping appraisal. Specifically, CC screening should be performed periodically for protection motivation. Table 1 presents examples of scripts and scenes in SeDAR^®^ from the HE agreement and women’s perspectives guided by the PMT constructs.

**Table 1:** The development of e-health SeDAR^®^ from the health experts (n = 12) agreement through the Focus Group Discussion (FGD) and women’s views (n = 7) through the in-depth interviews (IDI) based on the Protection Motivation Theory (PMT).

PMT constructs	^1^FGD health expert agreement / ^2^IDI women’s view	Scene (Minutes)	Example(s) characters and scripts in video
**Perceived vulnerability**	^2^ Emotional distress as a result of being diagnosed with cervical cancer	Prologue (minutes: 00:27)	ANA (main character) *Sometimes our lives will always be tested*. *I am confused and restless*. *Maybe this is destiny*
	^1^ Asymptomatic at the early stages makes women unaware the abnormality of cervical cells	Scene 6 (minutes: 04:32)	GRANDMOTHER (Ana’s mother in law) *It is important to do screening*. *Because at beginning*, *we didn’t see and realize the changers*. *But over the time*, *the symptoms spread*.
	^1^ The main risk factor for cervical cancer is persistent or repeated human papillomavirus (HPV) infection. ^1^Cervical cancer is a slow-growing disease	Scene 9 (minutes: 07:22) Scene 9 (minutes: 07:37)	DOCTOR *Main risk factor for cervical cancer is persistent infection from the human papillomavirus (HPV)*. *This continuous infection can lead to changes in the cervix*. *But this process will takes a long time*. *Perhaps around 10 to 20 years*.
**Perceived severity**	^2^ Interferes with the reproductive system	Scene 4 (minutes: 02:51)	ANA *Dear*! *I’m bleeding again*. *It’s not time for menstruation yet*.
	^1^Early detection and treatment is more effective if women attend screening at a younger age	Scene 12 (minutes: 11:30)	SHANTI (Ana’s best friend) *Don’t worry*! *If you get cervical cancer screening earlier*, *it will be easier for the doctor to diagnose and can prevent this cancer from spreading*. *Young people like us needs to do this screening early*.
**Fear (Treat Appraisal)**	^2^Did not do screening due to fear of being diagnosed with cervical cancer	Scene 7 (minutes: 05:24) Scene 12 (minutes: 11.20)	ANA *I don’t want to do screening*. *If I go to a check-up*, *who kwows what disease I will get*. *I don’t want to*! MIRA (Ana’s best friend) *I feel like doing the test*. *But what if it was me that got it* (cervical cancer)?
	^2^ Did not do the screening because of fear and embarrassment with the screening procedure	Scene 9 (minutes: 08:13)	ANA *Does the Pap smear hurt*? *I’m embarrassed to do it*.
	^1^ The screening procedure will cause some discomfort but is carried out by experienced staff to reduce discomfort	Scene 9 (minutes: 08:18)	DOCTOR *You will be asked to lie down*. *It may be a little uncomfortable*, *but our staff is experienced*. *So you don’t have to worry*.
**Perceived self–efficacy**	^2^ Alert about cervical cancer screening	Scene 6 (minutes: 04:27)	GRANDMOTHER *Yes*! *Its Pap smear*. *I*, *myself do it every three years*.
	^1^Feelings of discomfort and shame need to be overcome to ensure own health.	Scene 8 (minutes: 06:04)	ANA *Alright*. *I am doing this for the kid*. *But*, *I am embarrassed*. ZAYN (Ana’s husband) *Embarrassed*? *Honey*, *if we want to be healthy*, *we can’t have that mindset*.
	^2^ Get social support from husband and friends to increase confidence	Scene 6 (minutes: 04:32)	GRANDMOTHER *Your dad*, *when he was still alive*, *always reminded me to do screening*.
**Response efficacy**	^1^ Women who have been actively engaged in sexual intercourse need to undergo screening. Including unmarried women.	Scene 12 (minutes: 11:44)	SHANTI *Unmarried women that has been sexually active*, *also needs to do this screening*. *Young people like us*.
	^2^ Early detection will provide early treatment and indicate better quality of life.	Scene 10 (minutes: 09:36)	ZAYN *Is there a possibility for my wife to recover*, *doctor*? DOCTOR *If we start treatment immediately*, *the prognosis rate is high*. *So*, *let try our best*.
	^2^ Doing screening will make you feel relieved and more confident about your health status.	Epilogue (minutes: 12:16)	ANA *Now*, *I realize how important it is to do screening*. *There’s nothing to worry about something uncertain*. *I’m thankful that it’s all over*.
**Coping Appraisal**	^1^Screening helps early diagnosis and prevents the development of cervical cancer	Scene 6 (minutes: 04:32)	GRANDMOTHER *It’s (cervical cancer screening) important because we can’t see it*, *and people don’t realize how dangerous it is*. *But over time the symptoms spread*. *Please get Ana for check-up*. .
	^1^ Sexual active women should undergo cervical cancer screening using the HPV tests or Pap smear	Scene 9 (minutes: 07:45)	DOCTOR *Therefore*, *sexually active women are encouraged to undergo regular screening tests*. *There are two types of screening*. *One*, *is the Pap smear*, *which we do once every three years*. *Another test is HPV test*, *which we do once every five years*.
	^2^ Undergo screening for the benefit of own self, family and children.	Scene 8 (minutes: 05:46)	ZAYN *Darling*, *I love you so much*, *that’s why I want you to be healthy*. *Our children will be sad to see you sick*. ANA *Alright*. *I am doing this* (screening) *for the kid*.
**Protection Motivation**	^1^Regular cervical cancer screening should be done periodic, even if the previous results were negative.	Scene 9 (minutes: 08:04)	DOCTOR *We need to do screening regularly until we reach the age of 65*. *Regardless of whether the previous results are negative*.
	^2^ Change the mentality of women, even if they are healthy they need to be screened	Epilogue (minutes: 12:56)	ZAYN *Remember*, *educate yourself about everything*. *Especially when it concerns the cervical health*. ANA *Also*, *don’t forget to do your cervical cancer screening*. *Don’t wait until symptoms pop up*!

^1^ FGD = Focus group discussion

^2^ IDI = In Depth Interview.

### Validation

The SeDAR^®^ was validated to identify the expert agreement content validity index (CVI) among HE (n = 12) and ME (n = 5). The HE selected are the same expertise team from the FGD group, while for the ME the selection of experts is targeted by focusing on the diversity of backgrounds aimed at media, digital technology, information technology and broadcasting experts. The validation from expert was conducted from April 1 until April 20, 2023 with written consent from all participants. The CVI is an index of inter-rater agreement among experts and is a critical step in the development process of new instruments, and measures complex constructs in healthcare research [36]. The validation relies on an expert panel to evaluate the instrument elements and rate them based on their relevance and representativeness to the content domain. In this study, SeDAR^®^ underwent content validation using the CVI. The CVI was used because it was easier to understand, focuses on agreement of relevance rather than agreement per se, focuses on consensus rather than consistency, and provides both item and scale information [37].

The SeDAR^®^ expert validation questionnaire was adapted with permission from a previous study [38]. In the present study, the experts established the CVI to calculate the item-level CVI (I-CVI), and scale-level CVI-average (SCVI/Ave) and SCVI-universal agreement (SCVI/UA). The experts evaluated the video (concept, dramatic construction, rhythm, characters, drama potential, dialogue, visual style, target audience, relevancy, functionality, usability, efficiency) by rating each item on a 4-point Likert scale (1 = not relevant, 2 = somewhat relevant, 3 = quite relevant, 4 = very relevant). Items with a rating of 1 and 2 are considered invalid and items with a rating of 3 and 4 are considered valid. The CVI is computed by calculating the scale average by obtaining the consistency for item-level, I-CVI = Nr/N where Nr = number of experts voting (valid item 3 or 4) and N = total number of recruited experts [37,39]. The I-CVI range within 0 to 1. An I-CVI of 0.78 and SCVI/Ave ≥ 0.90 indicate good content validity [37]. The experts also commented on the video presentation.

### Usability

Subsequently, SeDAR^®^ was validated using purposive sampling among selected women (n = 11) with different socio-economic backgrounds (ethnicity, age, education level) and who were eligible for CC screening to achieve ecological validity. The usability validation was conducted from May 2 until May 15, 2023 with written consent from all participants. The video usability questionnaire was adapted from the previously developed and validated Video Engagement Scale (VES^®^) [40], which demonstrated high reliability (α = 0.93). The VES^®^ was developed to investigate patient-physician communication to engagement assessed of e-health video’s presumed correlates, e.g., perceived realism of the video and identification with the patient [40]. This VES^®^ evaluation form reliably evaluates viewers’ engagement according to five dimensional constructs (attention, entering a narrative world, identification, empathy, emotions) embedded in 15 statements. A higher VES^®^ score indicates better engagement. The women viewed the SeDAR^®^ video and completed the VES^®^ questionnaire, and commented on the video presentation.

## Results

**Fig 1:**
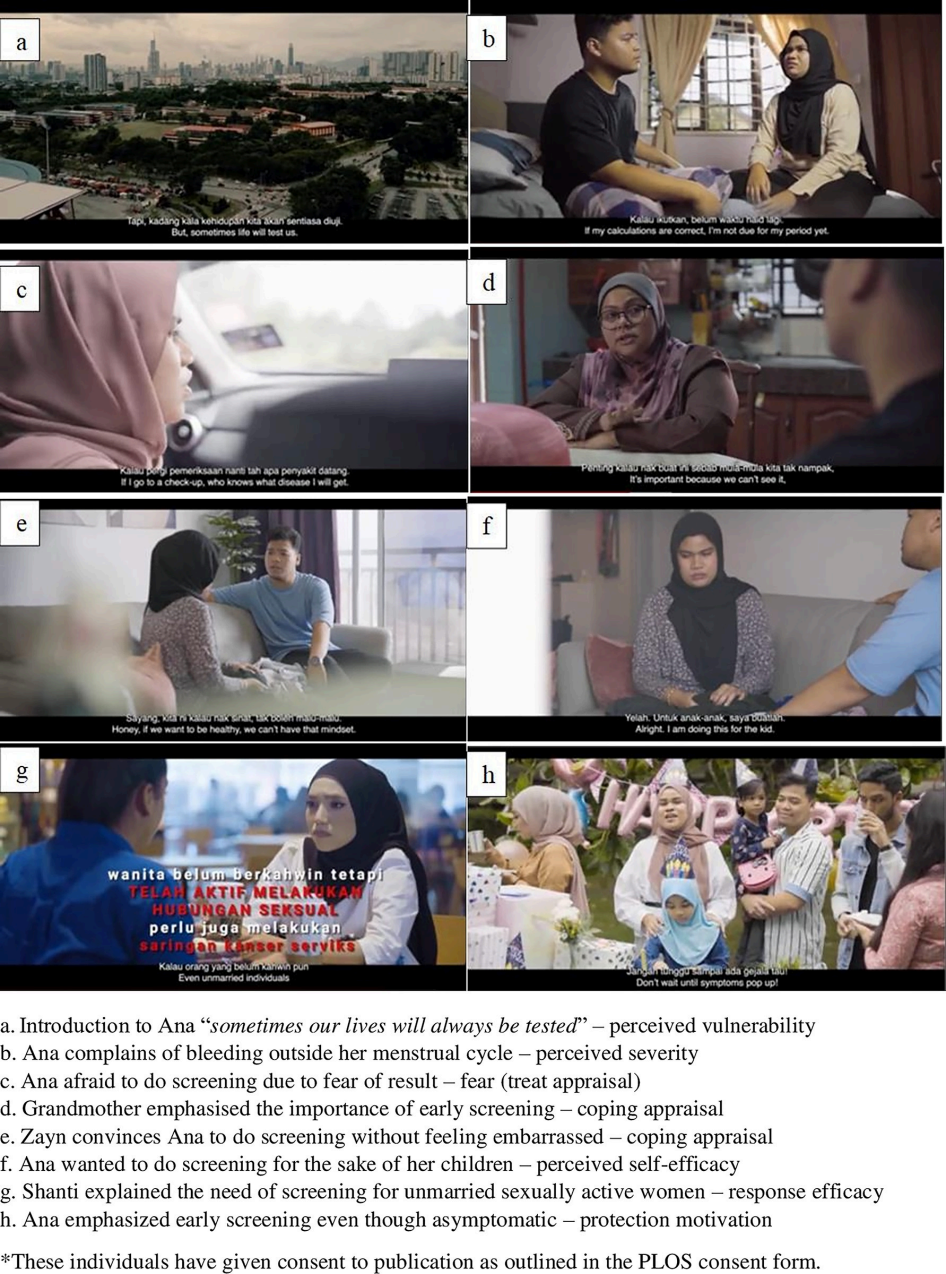
Scene in e-health video SeDAR^®^ based on protection motivation theory. *These individuals have given consent to publication as outlined in the PLOS consent form.

The 14-minute SeDAR^®^ short film is an entertainment-education motivational tool that was developed based on the HE perspective in conjunction with women’s and ME’s views. SeDAR^®^ was based on the true story of a CC survivor (“Ana”) who wanted to persuade women to not be afraid of CC screening and realise the importance of early periodic screening. The video contains five core segments comprising a prologue, 12 scenes, and an epilogue. The prologue establishes the main characters and topic, and scenes 1–3 establish the main character surrounded by her supportive family. The first core segment (scenes 4–6) highlights the emergence of symptoms and obtaining information. The second core segment (scenes 7–8) focuses on persuasion regarding the need for screening. The third core segment (scenes 9–12) addresses knowledge of CC, screening type, and screening eligibility. The epilogue concludes the story and reminds viewers of the key points, which include CC screening facilities. The overall content, storyline, and script were produced based on the seven PMT constructs. Fig 1 depicts an example of a scene in SeDAR^®^.

### Validation

We validated the expert agreement regarding SeDAR^®^ among HE and ME with the CVI. Twelve HE (two family medicine specialists, two obstetrics and gynaecology specialists, two public health specialists, two public health nurses, two community nurses, two health educators) and five ME answered the content validation questionnaire. The inter-expert CVI for both expert groups was 0.8–1, with an SCVI/Ave of 0.986 and 0.979, respectively, and an SCVI/UA of 0.900 and 0.897, respectively. Therefore, the results indicated good content validity [37]. Table 2 presents the distribution of the agreements. Two HE and one ME commented on the comprehensibility and presentation of the video as follows:

1. Refine the dialogue regarding unmarried women who want to undergo screening;
2. Insert text emphasising the free subsidised CC screening programme; and
3. Insert information or graphics when the dialogue explains facts.

**Table 2:** Expert agreement of content validity index of the e-health video SeDAR^®^ among health practitioner (n = 12) and media expert (n = 5).

Categories/subcategories	^a^Health practitioner (n = 12)			^b^Media expert (n = 5)		
	Experts agreement	I-CVI	UA	Experts agreement	I-CVI	UA
**Concept Idea**						
^a^ Relevant thematic content ^b^ Thematic content appropriate for objective	12	1.00	1	5	1.00	1
^a^ Coherent content with video objective ^b^ Assistance to learning	12	1.00	1	5	1.00	1
^a^ Video objective coherent with practice ^b^ Accessible	12	1.00	1	5	1.00	1
^a^ Assumptions exposed correctly ^b^ Script is useful	12	1.00	1	5	1.00	1
^a^ Comprehensible information ^b^ Attractive script	12	1.00	1	5	1.00	1
^a^ Sufficient Information	12	1.00	1			
^a^ Appropriate for the use of health professionals	12	1.00	1			
^a^ Proposes a change in behaviour	12	1.00	1			
**Dramatic Construction**						
^a^ Starting point has impact ^b^ Impactful starting point	11	0.917	0	4	0.80	0
^a^ Script interest grows ^b^ Interest in script grows	12	1.00	1	5	1.00	1
^a^ Nice script presentation ^b^ Sufficient number and duration of scenes	12	1.00	1	5	1.00	1
^a^ Scenes reflect stereotype / discrimination’ ^b^ Pleasant script presentation	10	0.833	0	5	1.00	1
**Rhythm**						
^a^ Scenes motive the next scenes ^b^ There is growing attraction with a dramatic curve	12	1.00	1	5	1.00	1
^a^ Tiring rhythm ^b^ Environment dynamism	12	1.00	1	5	1.00	1
^b^ Ways of presenting the appropriate scene				5	1.00	1
**Characters**						
^a^ Character empathy ^b^ Original character profile	10	0.833	0	5	1.00	1
^a^ Sufficient characters and situations ^b^ Characters with consistent values	12	1.00	1	5	1.00	1
**Drama Potential**						
^a^ Has emotion ^b^ The existence of expectation	12	1.00	1	5	1.00	1
^a^ Has surprises	12	1.00	1			
**Dialogue**						
^a^ Dialogue is natural ^b^ Each intervention motivates the next	12	1.00	1	5	1.000	1
^a^ Characters use appropriate language ^b^ There is action acceleration until the climax	12	1.00	1	4	0.800	0
^a^ Has an ending	12	1.00	1			
^a^ Relevant ending	12	1.00	1			
**Visual style**						
^a,b^ Symbols are easy to understand	12	1.00	1	5	1.00	1
^a,b^ Scenes reflect important aspects	12	1.00	1	5	1.00	1
**Target Audience**						
^a,b^ The content is related to the audience	12	1.00	1	5	1.00	1
^a^ Identification of the target audience with the problem	12	1.00	1			
^a^ Compatible language with the audience knowledge	12	1.00	1			
**Relevancy**						
^a^ Script illustrates important thematic aspects	12	1.00	1			
^a^ Relevant scenes for the target audience	12	1.00	1			
^a^ Script creates an abstract or revision	12	1.00	1			
**Functionality**						
^b^ This video invites viewers (eligible women) to change behavior towards cervical cancer screening.				5	1.00	1
^b^ Video gave positive results				5	1.00	1
**Usability**						
^b^ This video is easy to be use on various electronic platform and display in the health clinic				4	0.800	0
^b^ The concept is easy to learn and apply				5	1.00	1
^b^ Can be used by a health professional				5	1.00	1
**Efficiency**						
^b^ Proposed timing				5	1.00	1
^b^ Number of scenes are coherent to proposed timing				5	1.00	1
^b^ Characterization of the characters meets the proposed goals				5	1.00	1
^b^ Efficient and comprehendible communication between characters				5	1.00	1
**S-CVI/Ave**	0.986			0.979	
**S-CVI/UA**			0.900			0.897

Abbreviation: I-CVI = item-level content validation index, S-CVI = scale-level content validation index, S-CVI/Ave = the average of I-CVI scores across all items, S-CVI/UA = the average of UA scores across all items, UA = Universal Agreement.

**Fig 2:**
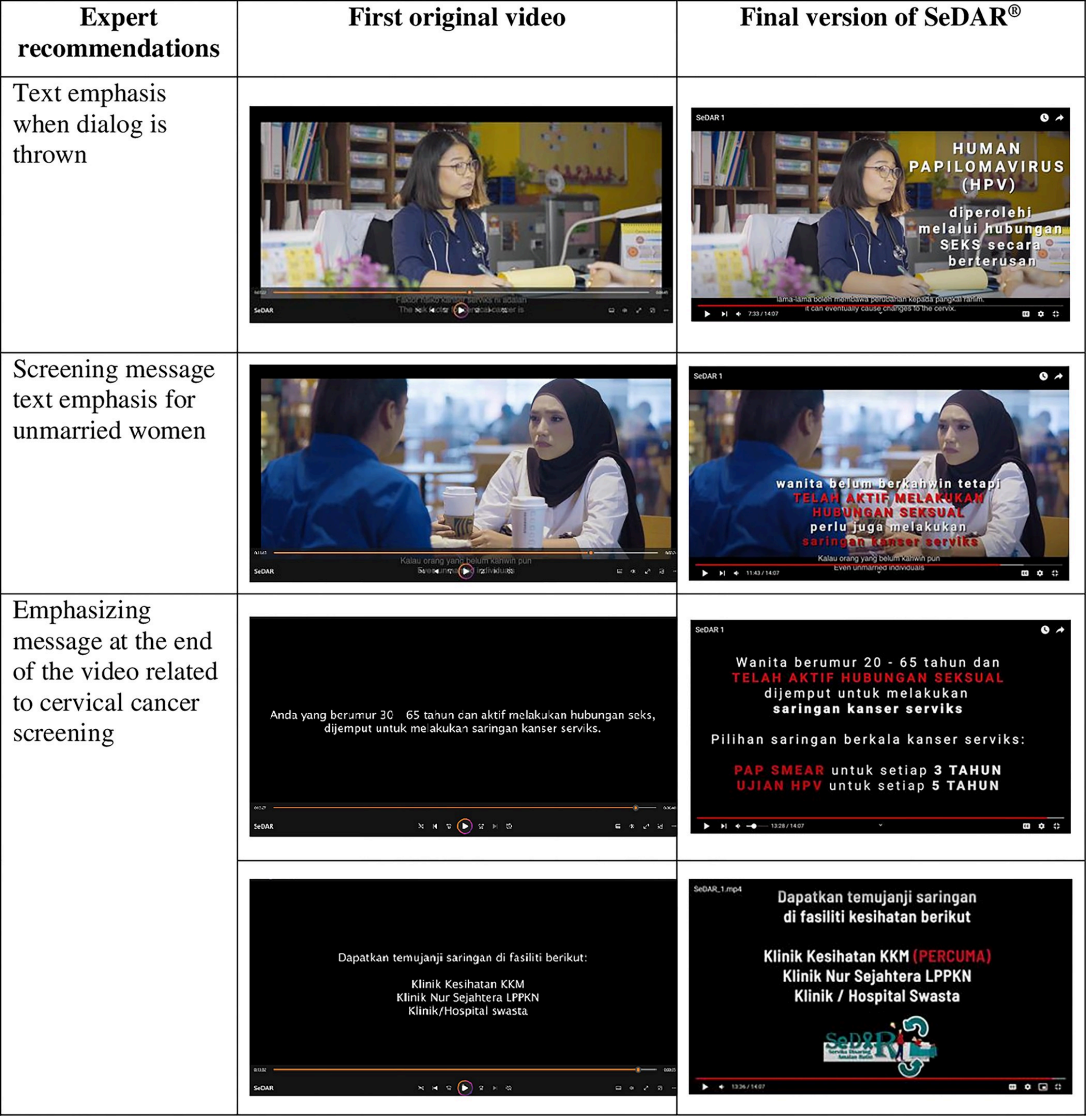
The improvements to e-health video SeDAR^®^ based on experts recommendations. *These individuals have given consent to publication as outlined in the PLOS consent form. The final version of SeDAR^®^ video is available on YouTube^©^ https://youtu.be/SbzLFvZa6gY.

Corrections were made based on these comments, and all 17 experts approved the corrected version of SeDAR^®^. Table 3 lists the experts’ comments on SeDAR^®^ and Fig 2 lists the improvements made.

**Table 3:** The experts’ comments on e-health video SeDAR^®^ and the improvement action.

Theme/Content	Expert Comment		Improvement Action (after discussion with the supervisor panel)
	Positive comments (no need improvement)	Comments/suggestions for improvement	
**Suitable for public viewing**	*Overall this video is suitable for public viewing*. (MS4)		None
**The content is clear**	*Content is very clear and an interesting videos*. *Simple but yet informative content*. (OGS2)		None
**Relevant content**	*Video content is relevant and informative*. *The script is easy to understand* (PHN1)		None
**Casual discussion**	*Casual discussions that give meaningful information*. (HE1)		None
**Screening for unmarried women**		*Conversation dialogue regarding the unmarried who want to undergo screening need to be refined*. (CN1)	Adding emphasis text related to "cervical cancer screening for unmarried women who have been sexually active"
**Text emphasis when dialog is thrown**		*Insert the info or graphics when the dialog explaining the facts* (MS1)	Added emphasis text related to cervical cancer screening when the doctor dialogue is thrown.
**Message emphasis at the end of the video**		*Insert text emphasizing on the free subsidized cervical cancer screening program*. (FMS2)	Added emphasis text related to "cervical cancer screening types and facilities that offer screening"

Abbreviations: MS = Media specialist; FMS = Family medicine specialist; OGS = Obstetric & gynecology specialist; PHS = Public health specialist PHN = Public health nurse; CN = Community nurse; HE = Health Educator.

### Usability

The corrected version of SeDAR^®^ was shown to the 11 women described earlier. SeDAR^®^ was shown via the WhatsApp^©^ application with a YouTube link for mobile phones. Soon after they had watched the video, the women answered the VES^®^ questionnaire via Google Forms. The score for every item answered ranged 5–7 (minimum score = 0, maximum score = 7), which indicated good agreement. Total scores ranged 87–98 (mean score: 92.90 [SD ±3.46]). Six of the woman also commented on the video comprehensibility and presentation as follows:

1. Fewer elements to make someone continue watching the video. But overall, it’s good;
2. Husband’s encouragement and support is very important;
3. The video is easy to understand. Simple but yet conveying;
4. The text pop-up during the doctor’s explanation is good;
5. This video is good in giving early exposure to the community about the need for early screening. It can change the stigma regarding the fear of screening; and
6. Nice video with good information.

Table 4 depicts the women’s VES^®^ scores and comments on SeDAR^®^ usability.

**Table 4:** The VES^®^ score and the usability of e-health video SeDAR^®^ among women (*n* = 11).

Respondent ID	Age	Ethnic	Education level	Total score (/105)^a^	Comment towards SeDAR^®^
**01**	30	Malay	Degree	92	“*Less elements to make someone stay on watching the video*. *But overall*, *it’s good*”
**02**	49	Chinese	Degree	95	*“I have experienced like Ana*, *worrying about check-up*. *So*, *I think this video brings a clear message to women and their partners about the importance of doing pap smear screening*. *Husband’s encouragement and support is very important too*.*”*
**03**	41	Malay	Secondary school	92	Not specified
**04**	32	Malay	Degree	98	*“The video is easy to understand*. *Simple but yet conveying”*
**05**	43	Malay	Diploma	90	Not specified
**06**	21	Punjabi	Diploma	97	Not specified
**07**	37	Indian	Degree	89	*“The text pop up during doctor’s explanation is good*. *In fact this video is touching emotional*, *full of information and relatable* (to women’s lives).”
**08**	42	Malay	Secondary school	91	*“This video is good in giving early exposure to the community about the need for early screening*. *It can change the stigma on fear of screening*.*”*
**09**	61	Malay	Secondary school	95	Not specified
**10**	26	Malay	Diploma	95	*“Nice video with good information”*
**11**	39	Malay	Secondary school	87	Not specified

^a^Mean (±SD) = 92.90 (±3.46).

## Discussion

We determined that the e-health motivational tool SeDAR^®^ has good content validity and acceptable usability. The positive feedback from health and media expert indicated that the video is clear, interesting, and informative, which is essential for engaging the audience. Furthermore, the video portrays casual discussions while providing meaningful information, which suggests that it effectively balances engagement and education. This approach enhances viewers’ understanding and interest in the subject matter. In video production, script writing and storyline development are crucial steps before filming [41,42]. Scriptwriting is the most important blueprint health educators must carefully consider, as it is constantly referenced throughout the project [43]. Hence, researchers should work together with the media production team to produce storylines that can achieve the video production objective by emphasising the motivational aspect based on the content developed based on the PMT. Thus, scripts and storyboards guide the creative process of video production.

We modified the video after receiving the experts’ feedback, where we added: (1) pop-up text emphasis during important dialogue, (2) text for the screening message for unmarried women, and (3) an emphasis message related to CC screening at the end of the video. Prior to the experts’ content validation, SeDAR^®^ was meaningful and was ready for testing. One woman provided positive feedback regarding the text pop-up depicted during the doctor’s explanation, where she felt it was good for increasing understanding related to the presented dialogue. Incorporating images and messages that create a compelling narrative and featuring prominent, memorable, and engaging visuals that align with the storyline can significantly enhance audience engagement [44,45]. Furthermore, including images on CC information in the second core segment (scenes 7–8), which focuses on persuasion regarding the importance of screening, effectively addressed perceived vulnerability and perceived severity as motivational factors.

Integrating the PMT framework into the SeDAR^®^ development was a crucial aspect in delivering effective information. Based on the PMT assumption, a person who feels the severity of a threat and feels at risk of being infected will demonstrate a high sense of threat appraisal towards the condition [31]. A previous cross-sectional study observed that women who had undergone CC screening perceived less fear, had higher response efficacy and self-efficacy, and perceived reduced response costs regarding CC screening. The experience of undergoing Pap smear screening could enable women to rationally address the benefits, undergo the screening with confidence, and become more motivated to undergo CC screening [32]. Ornelas et al. (2018) incorporated entertainment-education and narratives in their culture-based video and the results indicated a remarkable improvement in knowledge and intention for screening uptake, which can guide for similar video e-health interventions.

In healthcare research utilising videos as a study tool, assessing viewers’ engagement is important to determine external validity. An external validity known as ecological validity was used to evaluate whether SeDAR^®^ could be generalised to real-life settings [46]. For this reason, a validated VES^®^ was used due to its ability to assess the ecological validity of SeDAR^®^ and therefore contributed to the study rigor [40]. As the ecological validity of a study is a judgment and not a computed statistic, the high VES^®^ assessment scores proved the good ecological validity of the video. Furthermore, the HE and women expressed positive comments on the validity and usability of SeDAR^®^. Together with the CVI values, the findings indicated that SeDAR^®^ achieved both content and ecological validity for use in Malaysia.

SeDAR^®^ uses Malay as the communication language and has Malay and English subtitles. One limitation of this study is that Malaysian society is multilingual and speaks many languages, such as Mandarin, Tamil, and the Borneo native languages. Thus, the dissemination of knowledge among citizens who lack Malay and English language skills might be hindered. However, this culturally tailored e-health video will be able to reach more Southeast Asian viewers who have a similar Malay culture, such as those from Singapore, Indonesia, Thailand, and Myanmar. In fact, the use of subtitles in English, the world’s commonly used language, will be able to provide understanding to non-Malay viewers.

To the best of our knowledge, this is the first study on the development of an e-health tool that involved obtaining the views of both service providers and users regarding women’s motivation to undergo CC screening in Malaysia. The motivational interventions, which include community empowerment as a health education plan, can increase people’s power in influencing health determinants [47]. Previous studies that included community advisors in producing culturally tailored narrative videos [24] and local women in producing small media interventions (digital stories, fotonovelas, radionovelas) [17] efficaciously changed knowledge and intention to undergo CC screening. The present study is part of our response to the WHO global strategy to eliminate cervical cancer as a public health problem [48] to involve women in designing programmes related to CC health promotion. The combined information from providers and users will increase the impact of SeDAR^®^.

The scientific production of SeDAR^®^ with validity among expert and usability among woman proves its ability as an e-health tool based on motivation towards the uptake of CC screening. In relation to that, it can be an e-health tool that can be used by stakeholders including health authorities, health professionals, statutory bodies as well as non-governmental bodies such as women’s organizations. Furthermore, SeDAR^®^ videos can be used in the form of health promotion advertisements through national television programs, ministry of health’s official media social and dissemination through various platforms such as YouTube, Facebook, to reach more eligible women. Woman may watch SeDAR^®^ by themself, impart motivational message contained in this video and increasing self-efficacy for CC screening. The use of multiple technological mobile devices means that e-health tools are suitable in various settings, such as clinics and community organisations [24]. Health information through SeDAR^®^ video can always be disseminated through e-health tools and can reach marginalized women without boundaries including rural and urban areas, married and unmarried women who eligible for CC screening.

E-health has gained attention worldwide as it can overcome geographical barriers to promote health screening [49]. Engaging and easy to understand e-interventions appear effective for increasing the knowledge and uptake of CC screening [13]. A recent review identified a similar achievement when audio-visual aids were integrated into a 30-minute teaching intervention in rural India [20]. Additionally, a 15-minute Medical Aid film significantly improved participants’ knowledge regardless of age group, clinic site, primary language, education level, literacy, or access to healthcare providers (p < 0.0001) [22]. The implementation of e-health interventions in the developing countries and low- and middle-income countries (LMICs) might be achieved with organisational and technological infrastructural readiness [50]. Additionally, government, community, and health care provider involvement should be combined with public willingness to accept new, innovative e-health education tools. The use of the Internet of Things requires stable internet access. Technology has become more sophisticated and has been adopted even in remote areas that aim to become more developed. E-health information can reinforce positive health behaviours, especially among the younger generation [51]. Over the next decade, the world will move towards the WHO Shanghai Declaration on promoting health in the 2030 Agenda and on the full use of social innovation and interactive technology towards prioritising health policy [52].

## Limitations

One limitation of this study is that Malaysia has a multilingual society that speaks many languages. We have recruited various participants to appreciate the diversity of races in Malaysia. However, we could only include participant who spoke Malay and English, which are the two main Malaysian languages. Thus, the views of others who spoke other languages could not be captured during the validation and usability processes. Therefore, variety of participants with larger studies should be conducted in the future particularly for validation of new development product.

## Conclusion

We confirmed that the e-motivational video SeDAR^®^ is a valid and usable tool to be used in the Malaysian population. This entertainment-education instrument represents the perspectives of health care providers and users and was guided by the PMT framework [perceived vulnerability, perceived severity, fear (threat appraisal), response cost (coping appraisal), response efficacy, self-efficacy, protection motivation (intention)]. In particular, the study findings provide evidence about the validity and usability of SeDAR^®^ and support its use to increase the motivational aspects towards CC screening among eligible Malaysian women. The finding may also presented research contributions towards benefiting various stakeholders in the health care system including the women’s community, health professionals and service providers. SeDAR^®^ can be used as an e-health educational intervention tool to motivate women towards CC screening. Further research is needed to explore the effectiveness of SeDAR^®^ in promoting CC screening as compared to the traditional educational approach.

## Abbreviations

CC: cervical cancer
HPV: human papillomavirus
e-health: electronic health
PMT: protection motivation theory
SeDAR^®^: “*Serviks Disaring*, *Amalan Rutin*” (“Screened Cervix, Routine Practice”
CVI: content validity index
HE: health expert
ME: media expert
I-CVI: item-level content validation index
S-CVI/Ave: the average of I-CVI scores across all items
S-CVI/UA: the average of UA scores across all items
UA: Universal Agreement
